# Diagnostic difficulties in pediatric annular dermatoses

**DOI:** 10.1007/s10354-023-01019-3

**Published:** 2023-08-11

**Authors:** Doris Weiss, Philipp Weber, Amélie Hampel, Julia Tittes, Wolfgang Weninger, Tamar Kinaciyan

**Affiliations:** Department of Dermatology, Medical University of Vienna, Waehringer Guertel 18–20, 1090 Vienna, Austria

**Keywords:** Eosinophilic dermatoses, Eosinophilic annular erythema, Annular erythema of infancy, Wells’ syndrome, Eosinophile Dermatosen, Eosinophiles anuläres Erythem, Anuläres Erythem des Säuglingsalters, Wells-Syndrom

## Abstract

The polymorphic presentation of annular dermatoses in the pediatric population renders them a diagnostic challenge to the clinician. They include various distinct disease entities that can be vaguely categorized according to the age of onset. Herein, we report on a young girl with clinical characteristics of Wells’ syndrome, while histological findings favored the diagnosis of annular erythema of infancy (AEI). Although morphological and histological similarities do exist, AEI and eosinophilic annular erythema (EAE) of childhood are considered as distinct entities in the literature. Wells’ syndrome (WS) is an eosinophilic dermatosis and histologically characterized by eosinophilic dermal infiltration with the hallmark feature of “flame figures.” Based on this case, we discuss and review the differential diagnoses of annular dermatoses in children.

## Abstract (de)

Anuläre Dermatosen im Kindesalter stellen manchmal aufgrund ihres polymorphen Erscheinungsbildes eine Herausforderung im Klinikalltag dar. Sie umfassen verschiedene Krankheitsbilder, die grob nach den unterschiedlichen Altersgruppen eingeteilt werden können, in welchen sie sich erstmals manifestieren. In der vorliegenden Arbeit wird eine Patientin vorgestellt, bei der klinisch das Bild eines Wells-Syndroms vorlag, histologisch jedoch die „Flammenfiguren“ fehlten und der Befund für ein anuläres Erythem sprach. Das anuläre Erythem im Säuglingsalter („annular erythema of infancy“; AEI) und das eosinophile anuläre Erythem des Kindesalters („eosinophilic annular erythema“, EAE) existieren als distinkte Krankheitsbilder mit teils morphologischen sowie histopathologischen Ähnlichkeiten. Das Wells-Syndrom ist eine eosinophile Dermatose und zeigt histologisch ein dermales Infiltrat mit Eosinophilen und den charakteristischen „Flammenfiguren“. Basierend auf diesem Patientenfall werden die Differenzialdiagnosen der anulären Dermatosen im Kindesalter diskutiert.

## Case report

**Fig. 1:**
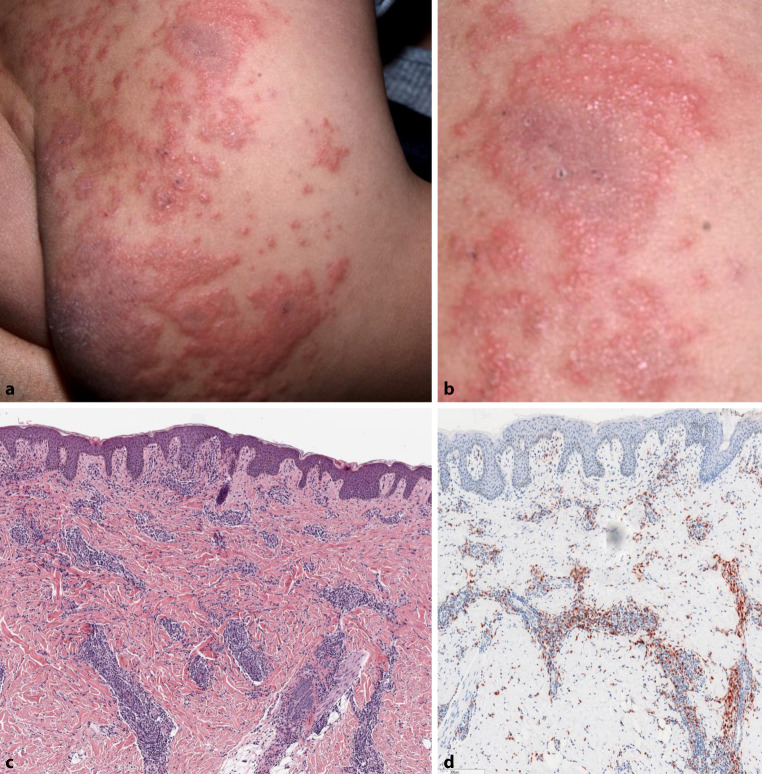
**a**, **b** Clinical feature overview and details: annular succulent papules and plaques with greyish-green center. **c** Histopathologic features of a punch biopsy: hematoxylin eosin (H&E) stain. Regular cornified epidermis. Perivascular and peri-adnexal lymphohistiocytic infiltrate in the upper and deep dermis. The scale bar is 300 μm. **d** Immunohistochemical staining of CD68 shows abundant macrophages within the infiltrate. The scale bar is 300 μm

A one-and-a-half-year-old girl presented to our pediatric dermatology clinic with erythematous papules and annular succulent plaques with a greyish-green center, confined to the right buttock (Fig. 1a, b). The parents had been applying a topical steroid ointment prescribed by a private practitioner for a few days, without efficacy.

The child’s medical history included trigonocephaly for which she had had surgical reconstruction, but was otherwise uneventful.

Our initial clinical differential diagnoses included Wells’ syndrome and eosinophilic annular erythema of childhood.

Histopathology, obtained from the border of the red to the greyish area of an annular lesion, revealed a superficial and deep perivascular and interstitial lymphocytic infiltrate with abundant CD68-positive macrophages (Fig. 1c, d).

White blood cell count and eosinophils were within the normal range. Eosinophilic cationic protein (ECP) and total serum IgE were both elevated, at 85.5 µg/L (normal value 13.3 µg/L) and 22.0 klU/L (age-adapted reference value < 10 kU/L), respectively. Family history of atopy and specific IgE antibodies to common inhalant and nutritive allergens were negative.

The lesions were treated with topical clobetasol propionate and occlusive dressing for 10 days, followed by once daily application that led to complete remission within 3 consecutive weeks. Since then, the patient has remained asymptomatic for the past 9 months without any further therapy.

## Discussion

Annular dermatoses in pediatric patients include a broad spectrum of differential diagnoses that can only be definitely diagnosed based on subtle clinical variations in conjunction with histopathologic features. In some cases, laboratory investigations are mandatory to rule out serious complications (Table 1).

**Table 1:** Differential diagnoses of annular erythema in children

	Diagnosis	Age	Pathogenesis	Clinical characteristics	Workup
Neonatal period	Neonatal lupus erythematosus	6–12 weeks	Maternal anti-SS-A Ab (95%) anti-SS-B Ab	Annular/polycyclic patches and plaques “Owl eyes“/“racoon eyes“ Photosensitivity Lesions resolve without scarring within 4–6 months (Ab degradation) Cardiac manifestations (AV block, cardiomyopathy)	*Clinical picture & serologic* testing in infant & mother *Histology:* Interface dermatitis, KC damage, perivascular, periadnexal lymphocytic infiltrate *DIF:* granular IgG deposition at dermo-epidermal junction
	Familial annular erythema	Usually soon after delivery	Autosomal dominant inheritance	Annular erythema with centrifugal spreading Very rare Positive family history is the key	*Histology:* superficial & deep perivascular infiltrate of marked neutrophils, few eosinophils & histiocytes
	Annular erythema of infancy (AEI)	Typically, within 3–11 months	Idiopathic	Recurrent annular erythematous plaques Slow spreading Resolving without sequelae within weeks	*Histology:* perivascular lymphocytic infiltrate with eosinophils
	Neutrophilic figurate erythema of infancy (NFEI)	Within days postpartum–13 months	Idiopathic	Similar to AEI No associated diseases/significant laboratory abnormalities	*Histology*: perivascular & interstitial neutrophils, nuclear dust with some lymphocytes ± eosinophils Absence of vasculitis
Infancy	Eosinophilic annular erythema (EAE)	Typically after the age of 1 year	Idiopathic	Annular erythematous lesions on trunk and extremities Recurrent and recalcitrant course over years	*Histology:* superficial and deep perivascular lymphohistiocytic infiltrate with abundant eosinophils
	Wells’ syndrome	Typically 1–9 years	Idiopathic Potential triggers: arthropod bites, vaccinations	Painfully infiltrated plaques with greenish hue Bullous form Systemic symptoms (fever, arthralgia, malaise)	Peripheral eosinophilia in > 50%, elevated ECP *Histology:* superficial & deep perivascular & interstitial eosinophilic infiltrate with “flame figures”
	Erythema annulare centrifugum (EAC)	Primarily adults affected, rare in children	Idiopathic EAC is a clinical reaction pattern, not a clinicopathological entitiy	Erythematous papules that enlarge centrifugally, ± scaling at the inner margin Individual lesions resolve within weeks Chronic course	*Histology:* perivascular lymphohistiocytic infiltrate, acanthosis, parakeratosis and spongiosis
Schoolchildren/​Pre-Teens	Erythema chronicum migrans (ECM)	Children after the age of 2	*Borrelia burgdorferi, B. afzelii, B. garinii*	“Bull’s eye”—expanding annular erythema with central clearing Manifests particularly in the head and neck region in children or with multilocular lesions Clinical picture determines diagnosis	*Serology:* unreliable in early manifestations or for monitoring purposes after successful therapy *Histology:* superficial & deep dermal lymphocytic infiltrate ± plasma cells and eosinophils
	Erythema marginatum (rheumaticum)	School children, teenagers, and adolescents	Acute rheumatic fever post-group A streptococcal infection	Recurrent centrifugally expanding erythematous annular patches & plaques Major Jones criterion for rheumatic fever (others include carditis, polyarthritis, chorea, and subcutaneous nodules)	*Histology*: interstitial and perivascular neutrophilic infiltrate without vasculitis. Erythrocyte extravasation in later stages
	Annular lichenoid dermatitis of youth (ALDY)	≥ 10 years of age	Idiopathic	Asymptomatic annular patches with an erythematous brownish border and central hypopigmentation Mostly on groin and flanks	*Histology:* lichenoid dermatitis limited to the tips of rete ridges with an intraepidermal lymphocytic infiltrate

*Ab* antibody, *DIF* direct immunofluorescence, *ECP* eosinophil cationic protein, *KC* Keratinocyte

Annular erythema of infancy (AEI) and eosinophilic annular erythema (EAE) of childhood are described as distinct, rare inflammatory conditions of infancy and childhood [1–5]. There are only a few cases previously published in the literature; therefore, the true incidence of these dermatoses is unknown and is likely underreported [1–7].

In 1981, Peterson and Jarratt originally coined the term AEI, for recurrent rapidly evolving arcuate and annular benign skin lesions, predominantly in infants from 3 to 11 months of age with spontaneous resolution within the first year of life. Histology consists of perivascular lymphocytes with scattered eosinophils [6]. Histiocytic infiltration has been described in HE staining but neither in that abundancy nor immunohistochemically (Fig. 1c, d; [7]). In contrast, EAE is thought to represent the morphological expression of a spectrum of eosinophilic dermatoses in older children that often follow a more recalcitrant clinical course, requiring immunomodulatory drugs. Histology shows a superficial and deep, predominantly perivascular, lymphocytic infiltrate with abundant eosinophils without “flame figures,” which are characteristic, but not pathognomonic, for Wells’ syndrome [1, 3, 8]. The only differences between AEI and EAE are the age of disease onset and the eosinophil count in histology (some in AEI and abundant in EAE), which results in a more transient rash in AEI or recalcitrant in EAE [3, 8].

Wells’ syndrome is a rare pruritic inflammatory skin disease of unknown etiology with several morphologic variants that have been described in recent years (papulonodular, papulovesicular, plaque-type, urticarial, or bullous). It classically presents as localized erythematous swelling (hence the term eosinophilic cellulitis), and subsequently progresses to indurated plaques with a characteristic green-greyish hue in the central part of the plaque.

In the present case, clinical findings were primarily compatible with Wells’ syndrome. The lack of eosinophils in histology, however, suggests otherwise, leaving room for debate. The natural tissue lifespan of eosinophils ranges from 2 to 5 days.^7^ Treatment with topical steroids downregulates interleukin (IL)-5, which is crucial for the recruitment and survival of eosinophils in tissue [9]. In hindsight it can only be speculated whether prior topical steroid treatment could obscure histopathologic findings (i.e., eradicate all tissue eosinophils).

Elevated levels of ECP in the absence of peripheral eosinophilia as well as the clinical presentation of annular succulent plaques with a greyish-green center (Fig. 1a, b) indicate eosinophil activation.

The age of onset after the first year of life would suggest EAE as the favorable diagnosis, while Wells’ syndrome does not have an age-specific predilection and is even considered to be rare in children [3, 8]. From our own experience, we know that Wells’ syndrome is probably underdiagnosed in many patients due to its polymorphic picture, as we have seen and successfully treated three children alone in the past 2 years. As the etiology is elusive in many cases, we propose that eosinophilic annular dermatoses in children might pose an academically unsatisfactory diagnostic dilemma. It remains to speculate whether they are different presentations of an identical pathophysiological process that lie within a spectrum and should not be exclusive of one another.
